# Association between triglyceride glucose index, coronary artery calcification and multivessel coronary disease in Chinese patients with acute coronary syndrome

**DOI:** 10.1186/s12933-022-01615-4

**Published:** 2022-09-16

**Authors:** Jiayu Wang, Xianwei Huang, Caihua Fu, Qiping Sheng, Ping Liu

**Affiliations:** 1 Department of Cardiology, The Second Hospital of Shandong University, Shandong University, No. 247, Beiyuan Road, Jinan, 250033 Shandong People’s Republic of China; 2 Department of Emergency, The First Affiliated Hospital of Xiamen University, Fujian, 361003 China; 3 Department of Cardiology, Jinan Central Hospital Affiliated Shandong University, Shandong, 250013 China

**Keywords:** Triglyceride glucose product index, Coronary artery calcification score, Acute coronary syndrome, Multivessel disease, Coronary computed tomography angiography

## Abstract

**Background:**

Multivessel coronary disease (MVCD) is the common type of coronary artery disease in acute coronary syndrome (ACS). Coronary artery calcification (CAC) has been confirmed the strong predictor of major adverse cardiovascular events (MACEs). Several studies have validated that triglyceride glucose (TyG) index can reflect the degree of coronary calcification or predict MACEs. However, no evidence to date has elucidated and compared the predictive intensity of TyG index or/and coronary artery calcification score (CACS) on multi-vascular disease and MACEs in ACS patients.

**Methods:**

A total of 935 patients, diagnosed with ACS and experienced coronary computed tomography angiography (CCTA) from August 2015 to March 2022 in the Second Hospital of Shandong University, were selected for retrospective analysis. The subjects were divided into TyG index quartile 1–4 groups (Q1-Q4 groups), non-multivessel coronary disease (non-MVCD) and multivessel coronary disease (MVCD) groups, respectively. The general data, past medical or medication history, laboratory indicators, cardiac color Doppler ultrasound, CACS, and TyG indexes were respectively compared among these groups. The ROC curve preliminarily calculated and analyzed the diagnostic value of TyG index, CACS, and the combination of the two indicators for MVCD. Univariate and multivariate logistic regression analysis discriminated the independent hazard factors for forecasting MVCD.

**Results:**

Compared with the lower TyG index and non-MVCD groups, the higher TyG index and MVCD groups had higher values of age, smoking history, waist circumference, systolic blood pressure, low-density lipoprotein cholesterol(LDL-C), fasting blood glucose and glycosylated hemoglobin, and CACS, but lower values of high-density lipoprotein cholesterol(HDL-C) (all *P* < 0.01). Coronary artery calcification is more common in the left anterior descending artery. Compared with non-MVCD, each unit increase in TyG index was associated with a 1.213-fold increased risk of MVCD. Logistic regression analysis adjusted for potential confounders indicated that TyG index is an independent risk factor for MVCD. With the increase of TyG index, the incidence of MACEs, apart from all-cause death, cardiac death, unexpected re-hospitalization of heart failure, recurrent ACS or unplanned revascularization, and non-fatal stroke in coronary artery increased (*P* log-rank < 0.001).

**Conclusion:**

TyG index could completely substitute for CACS as a reliable, practical, and independent indicator for predicting the severity and prognosis of MVCD in patients with ACS.

## Introduction

Arteriosclerotic cardiovascular disease (ASCVD), especially acute coronary syndrome, is the primary cause of mortality and disability worldwide [1, 2]. Acute coronary syndrome (ACS), including unstable angina pectoris (UAP), non-ST-elevation myocardial infarction (NSTEMI) and ST-elevation myocardial infarction (STEMI) [3], is considered the most severe clinical condition of ASCVD. The mortality of ACS patients after 1 year is approximately 15%, and the cumulative mortality after 5 years is as high as 20% [2].

ACS patients are often complicated with multivessel coronary disease (MVCD), the advanced type of coronary artery disease, have more risk factors, may undergo more revascularization intervention and bypass surgery, have a higher incidence of MACE events and worse long-term prognosis than other patients with single vessel coronary disease or non-MVCD [4, 5]. The underlying pathophysiology for ACS is characterized by 3 most common pathological types: rupture, erosion, and calcified in atherosclerotic plaque [3]. Coronary artery calcification (CAC) is a widely recognized feature of advanced atherosclerosis and often reflects the existence of coronary disease [6–9]. Regardless of risk factors or symptoms, multicenter studies of atherosclerosis research have shown that CAC is a powerful predictor of major adverse cardiovascular events (MACEs) and provides clinical information that goes beyond the traditional risk factors for ASCVD-related morbidity and mortality [10–14]. Coronary calcification is only a pathological phenomenon, and the influence of the lesion degree of CAC on ASCVD outcome needs to be incarnated by calcification score (CACS) [13, 15]. As a surrogate marker of plaque burden and an independent predictor, the prognostic utility CACS has been established in the future cardiovascular events [16]. Patients with more severe coronary calcification more likely to have higher plaque burden, more adverse plaques, severe coronary artery stenosis, and MACEs [13, 15, 16].

Although CACS is a noninvasive quantification method of CAC using computed tomography, it requires a high dosage of contrast agents, which may cause kidney damage or allergic reactions. Similarly, invasive coronary artery examination for finding the severity of coronary artery disease, including coronary angiography (“gold standard”), optical coherence tomography (OCT), and intra-vascular ultra sound (IVUS), requires the use of contrast agent injection, imaging guide wire or invasive catheter intervention, and other auxiliary equipment, which is not only time-consuming and expensive, but also undertake the risk of contrast nephropathy and allergy, and various operations complications after hospitalization, which limited the application of these technologies for identifying coronary morphology and assessing calcified plaque to a considerable extent. Therefore, to find convenient biomarkers is of great significance for the early detection and forecast of ASCVD.

Recently, as a reliable surrogate for insulin resistance, TyG index has been certified to be an independent predictor for the progression of coronary artery calcification by Won KB, et al. [13, 15]. Moreover, Wang L, et al. held that TyG index also associates with coronary MACEs and can independently predict the prognosis of ASCVD [17, 18]. The TyG index was originally studied as a marker of identifying insulin resistance (IR) [15, 19, 20]. It is first proposed by Simmental-Mendía and Guerrero-Romero that TyG index is a composite indicator composed of TGs and FBG, and calculated by Ln (fasting triglyceride [mg/dl] × Fasting blood glucose [mg /dl]/2) [19]. Some studies have confirmed that compared with several lipid ratios such as low-density lipoprotein cholesterol (LDL-C)/high-density lipoprotein cholesterol (HDL-C), non HDL-C/HDL-C, and apolipoprotein B/apolipoprotein A1, visceral obesity index, and lipid accumulation products, TyG index had more significant correlation with homeostasis model assessment (HOMA) in both long-term and short-term coronary events after ACS, independent of diabetic status [20, 21]. Increasing evidence shows that TyG index can substitute for hyperinsulinemic-euglycemic clamp (HIEC) and HOMA to assess IR, and has been identified as an essential mediator of metabolic disorders, type 2 diabetes (T2DM) and ASCVD [20, 22]. However, to our knowledge, so far, there is no report on the relationship between TyG, CACS, and the severity of MVCD for Chinese with acute coronary syndrome.

This study selected 935 ACS cases hospitalized in the Second Hospital of Shandong University. We performed a 128-MSCT scan on the participants and calculated the calcification score (CACS) of these patients suffered from ACS with single-vessel and multivessel coronary artery diseases through image analysis software, and retrospectively analyzed the predisposing factors, clinical and biochemical indicators, CACS, TyG index and the correlations among parameters and variables in these patients, in order to explore the diagnostic value and predictive intensity of calcification score and TyG index for the degree of coronary artery disease and cardiovascular MACEs, so as to find a convenient clinical index to predict coronary lesion severity and ASCVD outcomes. It is helpful for the early identification, diagnosis, and treatment of patients with MVCD.

## Methods

### Study population

This study was a single-center, retrospective, observational cohort study. From August 2015 to March 2022, 935 consecutive patients with ACS in the Second Hospital of Shandong University were selected, who ware completed the coronary computed tomography angiography (CCTA) within 1 week before admission or during hospitalization and underwent coronary angiography in the hospital. Sex analysis showed that there were 645 males and 290 females. The oldest was 97 years old, the youngest was 27 years old, and the average age was 64.97 ± 13.68 years. According to the coronary angiography, the patients were divided into the multivessel coronary disease (MVCD) group and the non-multivessel coronary disease (non-MVCD) group. Exclusion criteria: (1) patients with severe liver and kidney dysfunction; (2) patients with severe arrhythmia, cardiomyopathy, valvular disease, and congenital heart disease; (3) patients with severe infection, thyroid function disease, hematologic disorders, autoimmune disease, tumor; (4) patients with systemic disease severe diseases, and unstable vital signs; (5) recent severe trauma or major surgery; (6) pregnant or lactating women; (7) severely missing relevant information. Most importantly, the study was approved by the Ethical Committee of The Second Hospital of Shandong University and consecutive individuals were retrospectively enrolled into this study, who suffered from ACS. Additionally, written/oral informed consent was also obtained from all participants.

### General information of the subjects

General information of the subjects was collected in the study, including gender, age, body Mass Index (BMI), blood pressure, heart rate, history of smoking and drinking, past medical history (chronic diseases such as hypertension and diabetes), and the application of lipid-lowering, antihypertensive, and hypoglycemic drugs. Collected the morning fasting venous blood and inspected related laboratory indicators, including white blood cell (WBC), fasting blood-glucose (FBG), total cholesterol (TC), triglyceride (TG), low density lipoprotein cholesterol (LDL-C), high-density lipoprotein cholesterol (HDL-C), creatinine (Cr), cystatin C (Cys C), homocysteine (Hcy), uric acid (UA), brain natriuretic peptide (BNP), troponin I (TnI), D-dimmer, aspartate aminotransferase (AST), and alanine aminotransferase (ALT). Within 24 h after admission, cardiac color Doppler ultrasound measures the left atrial diameter (LA), left ventricular end-diastolic volume (LVED), left ventricular ejection fraction (LVEF), and the ratio of early diastolic mitral valve blood flow velocity to early diastolic mitral valve annulus velocity (E/E’).

### CT acquisition and image analysis

After independent assessments were made, a final CCTA diagnosis was obtained based on consensus interpretation. CACS was calculated in the software through the CT angiography of coronary arteries plain scan level.

**Fig. 1:**
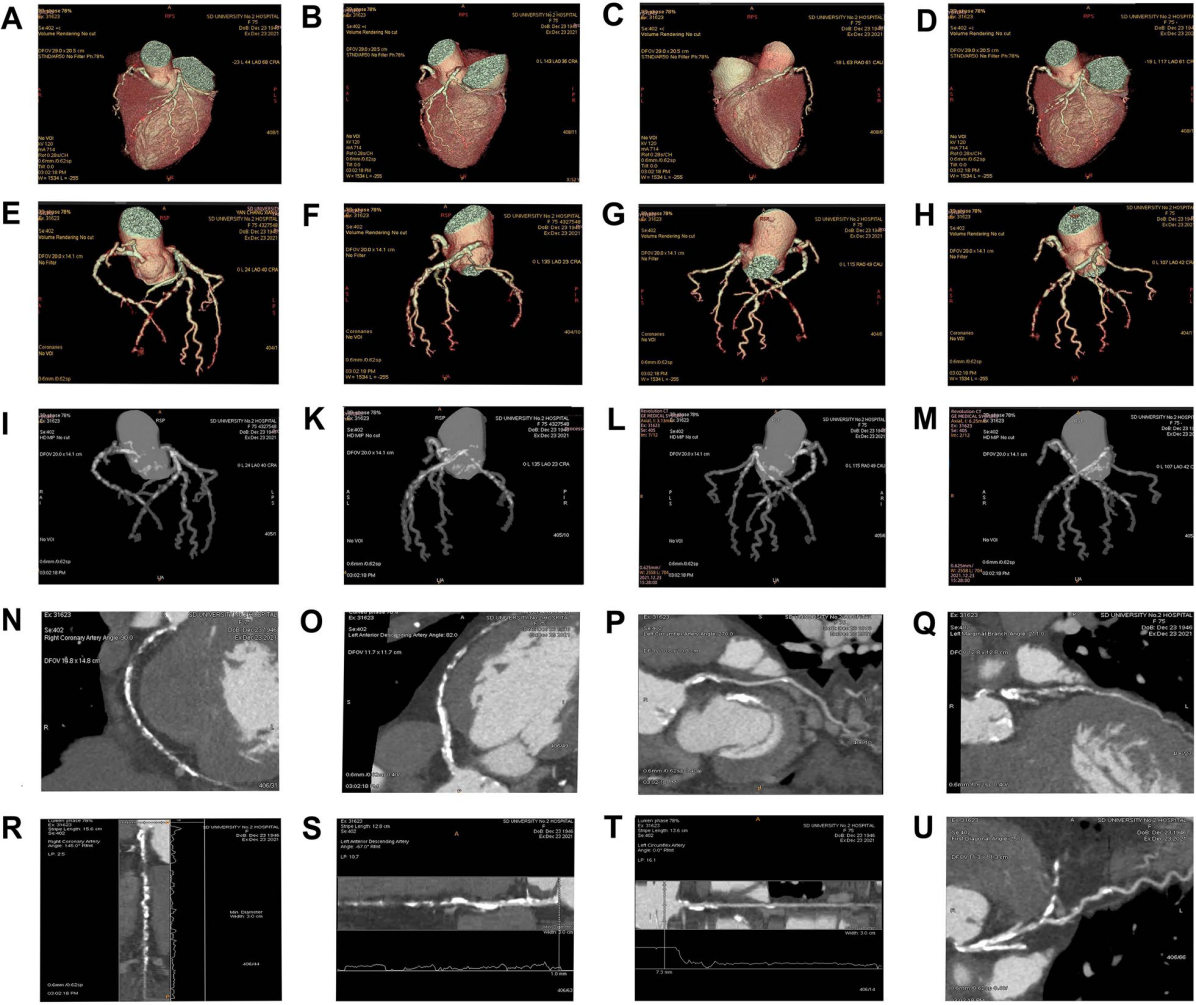
CTA images of a 75 year old woman with severe coronary calcification and disease. Different coronary CTA three-dimensional reconstruction techniques were used: **A**–**D**, VRT (volume rendering technique) for the whole myocardium and coronary arteries. **E**–**H**, VRT for coronary vascular trees. **I**–**M**, MIP (maximum intensity projections) for coronary vascular trees. **N**–**U**, CPR (curved planner reconstruction) for the degree of coronary stenosis and calcification in each large coronary branch: right coronary artery (**N** and **R**); left anterior descending branch (**O** and **S**); left circumflex branch (**P** and **T**); left marginal branch (**Q**); first diagonal branch (**U**)

*Adopted angiography technology methods*: All of coronary angiograms, using multi-slice spiral CT scanner (128-MSCT, Discovery CT 750HD, GE Healthcare, Waukesha, WI, USA), combined use of American Meorao CT high-pressure double barrel syringe, and GE Onepak hypotonic non-ionic contrast medium with 350mgI/ml iodine concentration [Iohexol injection, Onepak, GE Pharmaceutical (Shanghai) Co., Ltd.], were independently estimated by two independent angiographers double-blind to the random assignment and the clinical information of subjects [23]. Preparation before scanning: the patient fasted for 4–6 h and signed the informed consent form. Fully communicate with the patient before examination, hold breath 3 times, and strictly observe the changes in the center rate during breath-hold process. Scanning parameters: the scanning layer thickness is 0.625 mm and the acquisition matrix is 512 mm × 512. Display matrix 1024 × 1024. The tube voltage is 120kv and the tube current is 550 mA for spiral volume acquisition and scanning. The chest was positioned on the frontal and lateral positioning film. The scanning range from 1 cm below the tracheal ridge to 2 cm below the diaphragm is the "scanning area" to cover the whole heart. The contrast agent was 350 mgI/ml, 90 ml contrast medium and 35 ml normal saline, which was rapidly injected through the right elbow vein at a rate of 5 ml/s. The delay monitoring time of trigger point of intelligent tracking technology is 10 s, and the threshold is 100HU. The post-processing of CT image data: transfer the collected original image data information to GEADW4.6 image post-processing workstation, and use the relevant post-processing and reconstruction software package to carry out multi-planar reconstruction (MPR), Maximum Intensity Projection (MIP), surface shaded display (SSD), volume reproduction (VR), and surface reconstruction (CPR) technologies to optimize the acquisition of relevant image data, and cooperate with cutting, rotation, and 3D domain removal tissue synthesis technology to make the corresponding anatomical structures of blood vessels and surrounding tissues more clear and to reconstruct image information. According to the heart rate, after 40%, 45% and 75% of the original axial data images without motion artifacts in the RR interval are selected for processing, the selected phase data collected by the front door control is 75%. Image data reconstruction methods mainly include volume reconstruction technology and surface reconstruction technology (Fig. 1). Through observing the collected axial cross-sectional image data, the preliminary screening of coronary artery lesions is carried out, and then the lesions are displayed in three-dimensional by using different image post reconstruction technologies. Through CPR and VR technology, the coronary artery and small branches can be displayed quickly and stereoscopically.

Significant coronary artery stenosis was defined as at least 50% stenosis in any of the major vessels (left main coronary artery, left anterior descending artery, left circumflex artery, or right coronary artery). The severity of coronary disease in each subject was quantified by the number of coronary arteries involved. Single-vessel disease was defined as at least 50% stenosis in one major coronary artery [23]. Multivessel disease was defined as at least two major vessels (≥ 2 mm diameter) from different territories with lesions deemed angiographically significant (≥ 50% stenosis) [4, 24]. A 50% or more reduction in the internal diameter of the left main coronary artery was considered to be three-vessel disease [23].

### Description of relevant CT indicators

**Fig. 2:**
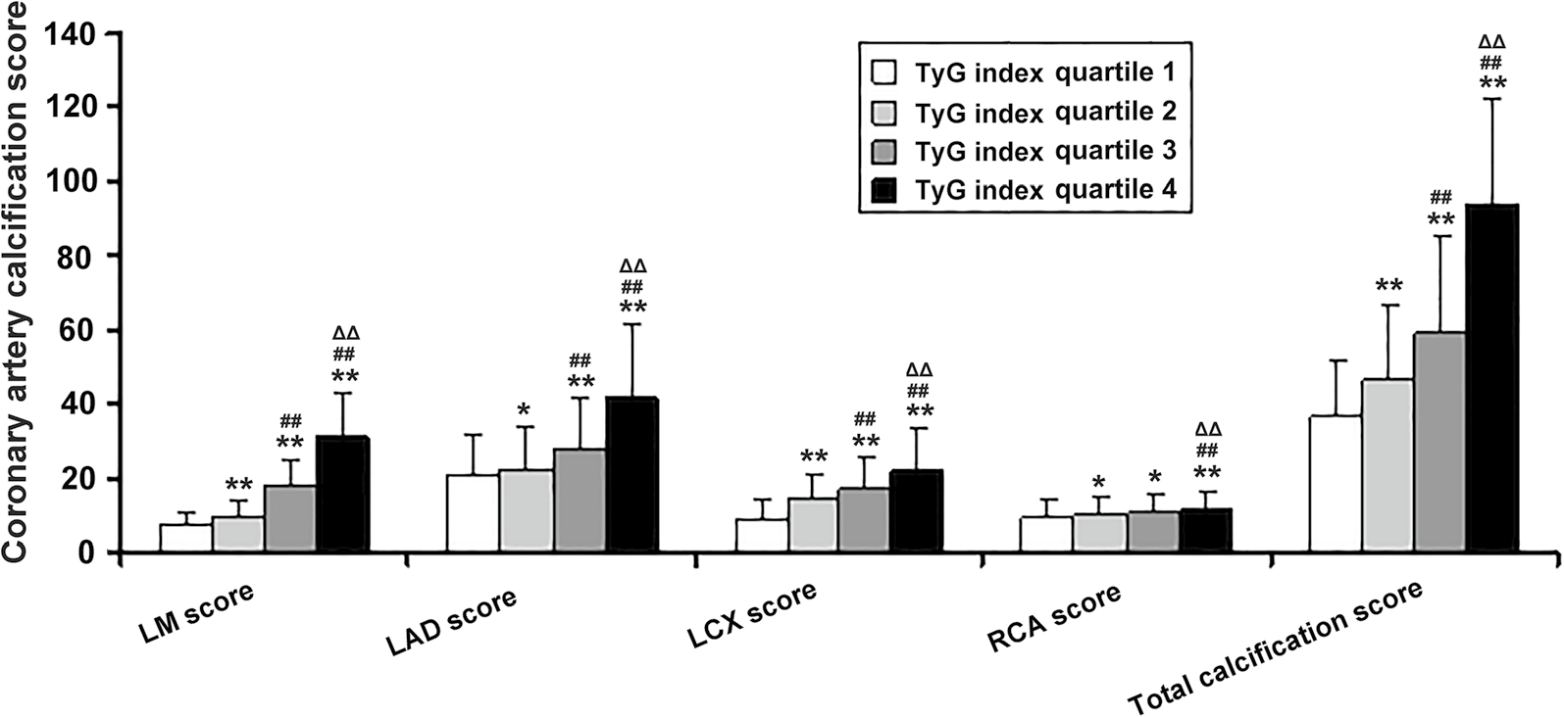
Comparison of coronary calcification scores in each quartile array of TyG index. vs. TyG index quartile 1 group. *P < 0.05, **P < 0.01; vs. TyG index quartile 2 group, #*P* < 0.05, ##*P* < 0.01; vs. TyG index quartile 3 group, ∆*P* < 0.05, ∆∆*P* < 0.01

The subject’s CT angiography of coronary artery images through the AW VolumeShare 5 system was checked, and the corresponding chest scan images were selected. Two senior attending physicians who have worked in the imaging department for more than 10 years read each in the SmartScore software in CardIQ Xpress. One layer of plain scan image to mark the calcification part, and finally, the CACS result calculated by Volume-130 shall prevail (Figs. 1 and 2). TyG index = Ln[fasting serum triglycerides (mg/dl) × fasting blood glucose (mg/dl)/2] [19]; where triglycerides 1 mmol/L = 88.6 mg/dl, fasting blood glucose 1 mmol/L = 1/18 mg/dl.

### Statistical analysis

SPSS software, version 19.0 (SPSS, Inc., Chicago, IL, USA) was applied for statistical analysis of the data. Continuous variables are shown as mean ± standard deviation (SD) or median (inter-quartile range), and t-test and F-test to compare the statistical difference between the mean values of two or more independent variables. Mann–Whitney U test was used to compare abnormally distributed data, and the results were expressed as median and inter-quartile range. Enumeration data was expressed as a percentage (%), and the chi-square test was applied to compare categorical variables. The ROC curve analyzed the diagnostic value of CACS, TyG index, and the combination of the two variables for MVCD, calculated the area under the curve, and determined the sensitivity and specificity of forecasting MVCD. Univariate and multivariate logistic regression analysis demonstrated the correlation between some independent hazard factors including CACS, TyG index and coronary multivessel disease. *P* < 0.05 considered the difference to be statistically significant.

## Results

### Baseline characteristics

The mean age of 935 subjects at baseline was 64.97 ± 13.68 years, and 68.98% of the subjects were men (n = 645). The included subjects were divided into four groups (Q1-Q4 groups) according to the level of TyG index. The clinical characteristics, biochemical and cardiac Doppler ultrasound parameters of the subjects are shown in Table 1. Patients with a high TyG index were more likely to be the male sex, however, there was no statistical difference among the groups (Table 1). General information and vital signs: age, systolic blood pressure (SBP), diastolic blood pressure (DBP), pulse pressure difference (PPD), heart rate (HR); anthropometric measurements: weight, body mass index (BMI), waist circumstance (WC); Laboratory indicators: white blood cell (WBC), uric acid (UA), creatinine (Cr), cystatin C (Cys C), fasting blood glucose (FBG), glycosylated hemoglobin A1c (HbA1c), total cholesterol (TC), triglyceride (TG), low density lipoprotein cholesterol (LDL-C), troponin I (TnI), D-dimmer, homocysteine (Hcy), and liver functions (aspartate aminotransferase, AST; alanine aminotransferase, ALT) were elevated, whereas high density lipoprotein cholesterol (HDL-C), and antihypertensive, hypolipidemic, and hypoglycemic drugs application were decreased in higher TyG index groups. Meanwhile, there was no significant difference in echocardiographic parameters (left atrial diameter, LA; left ventricular diastolic diameter, LVED; left ventricular ejection fraction, LVEF; ratio of early diastolic mitral flow velocity to early diastolic mitral annular motion velocity, E/E’) and alcohol consumption among Q1-Q4 groups. Moreover, the high TyG index group had more hypertension, diabetes, and smoking except for the alcohol consumption (Table 1). With the increase of TyG index, the total CACS and the CACS of each coronary artery branch [left main artery (LM), left anterior descending artery (LAD), left circumflex artery (LCX), right coronary artery (RCA)] in each patient also gradually increased (all *P* < 0.05) (Fig. 2).

**Table 1:** Baseline characteristics of subjects stratified by the TyG index quartiles

Project	TyG index quartiles				*F* or *χ*^*2*^	*P*
	Q1 (n = 226)	Q2 (n = 239)	Q3 (n = 224)	Q4 (n = 246)		
General information						
Age (year)	60.56 ± 12.31	63.81 ± 12.78	62.50 ± 14.05	68.22 ± 13.40	36.45	0.000
Male sex [case (%)]	151 (66.81%)	155 (64.85%)	167 (74.55%)	176 (71.54%)	6.37	0.095
SBP (mmHg)	136.75 ± 18.91	141.98 ± 20.48	147.28 ± 20.30	155.45 ± 21.26	81.15	0.000
DBP (mmHg)	80.87 ± 11.23	80.15 ± 11.98	82.22 ± 12.11	82.24 ± 11.78	4.20	0.000
PPD (mmHg)	56.42 ± 16.68	62.12 ± 19.35	64.73 ± 19.57	72.49 ± 20.02	61.07	0.000
HR (bpm)	76.46 ± 13.89	77.12 ± 14.11	78.49 ± 14.82	78.77 ± 13.73	5.11	0.002
Past medical history						
Smoking [case (%)]	71 (31.41%)	9 5 (39.75%)	108 (48.21%)	139 (56.50%)	33.46	0.000
Drinking [case (%)]	54 (24.0%)	58 (26.4%)	59 (23.0%)	74 (31.8%)	3.00	0.391
Hypertension [case (%)]	127 (56.19%)	118 (49.37%)	153 (68.30%)	141 (57.32%)	17.33	0.000
Diabetes [case (%)]	50 (22.12%)	40 (16.74%)	75 (33.48%)	83 (33.74%)	26.13	0.000
Antihypertensive drugs [case (%)]	97 (42.92%)	84 (35.15%)	76 (33.93%)	65 (26.42%)	20.91	0.000
Hypoglycemic drugs [case (%)]	89 (39.38%)	86 (35.98%)	72 (32.14%)	68 (27.64%)	8.11	0.044
Hypolipidemic drugs [case (%)]	68 (30.08%)	38 (15.90%)	40 (17.86%)	38 (15.45%)	20.91	0.000
Anthropometric measurements						
Body weight	68.60 ± 7.80	69.42 ± 7.04	72.54 ± 8.43	75.62 ± 8.34	88.17	0.000
Body mass index (BMI)	25.63 ± 2.93	26.53 ± 3.19	27.88 ± 3.50	30.52 ± 3.89	209.84	0.000
Waist circumstance (WC)	88.48 ± 7.71	90.61 ± 7.56	95.19 ± 9.66	102.62 ± 10.66	251.79	0.000
Laboratory indicators						
WBC (×10^9^/L)	7.84 ± 2.95	8.16 ± 2.58	8.54 ± 3.04	8.85 ± 2.74	11.69	0.000
UA (umol/L)	321.18 ± 115.87	341.72 ± 109.45	362.91 ± 111.00	407.86 ± 140.68	50.34	0.000
Cr (umol/L)	75.89 ± 24.46	78.52 ± 31.61	78.71 ± 31.61	82.43 ± 33.18	4.31	0.005
Cystatin C (mg/L)	1.24 ± 0.64	1.26 ± 0.62	1.34 ± 0.71	1.79 ± 1.08	63.69	0.000
FBG (mmol/L)	5.46 ± 0.89	6.03 ± 1.25	6.84 ± 1.63	9.50 ± 3.03	41.32	0.000
HbA1c	6.21 ± 0.97	6.86 ± 1.46	7.56 ± 1.72	10.31 ± 2.87	43.17	0.000
TC (mmol/L)	3.97 ± 0.90	4.28 ± 1.08	4.38 ± 1.04	4.53 ± 1.20	17.86	0.000
TG (mmol/L)	0.88 ± 0.23	1.36 ± 0.31	1.96 ± 0.52	3.33 ± 1.26	86.17	0.000
LDL-C (mmol/L)	2.34 ± 0.78	2.63 ± 0.89	2.74 ± 0.96	2.75 ± 1.04	12.17	0.000
HDL-C (mmol/L)	1.51 ± 0.76	1.42 ± 0.53	1.19 ± 0.52	0.92 ± 0.43	14.75	0.000
BNP (pg/ml)	504.03 ± 324.15	586.78 ± 313.82	430.73 ± 308.46	714.76 ± 345.74	0.61	0.606
TnI (ng/ml)	10.66 ± 3.47	11.83 ± 4.60	10.57 ± 5.68	18.89 ± 10.89	32.11	0.000
D-dimer (ng/ml)	687.72 ± 501.04	393.80 ± 378.42	537.69 ± 409.94	513.67 ± 445.10	2.82	0.037
Homocysteine (umol/L)	14.76 ± 9.79	16.85 ± 16.34	15.67 ± 11.09	17.04 ± 9.74	3.19	0.023
AST(U/L)	98.18 ± 76.47	86.54 ± 64.57	81.87 ± 57.30	115.85 ± 82.89	9.98	0.000
ALT(U/L)	39.16 ± 36.45	30.80 ± 25.20	33.26 ± 30.20	39.12 ± 36.04	3.01	0.029
Echocardiography						
LA	36.48 ± 4.47	36.92 ± 4.95	36.84 ± 5.05	37.05 ± 5.05	0.87	0.458
LVED	48.79 ± 4.66	49.14 ± 5.13	48.95 ± 5.73	48.98 ± 4.714.22	0.25	0.865
LVEF	54.23 ± 8.61	55.26 ± 8.11	54.35 ± 8.78	53.84 ± 9.948.98	2.58	0.052
E/E’	9.16 ± 3.43	9.03 ± 3.71	9.28 ± 4.44	9.55 ± 4.11	1.96	0.118

*SBP* systolic blood pressure; *DBP* diastolic blood pressure; *PPD* Pulse pressure difference; *HR* heart rate; *LLD* Lipid-lowering drugs; *BMI* Body mass index; *WC* waist circumstance; *WBC* white blood cell; *Cr* creatinine; *UA* uric acid; *FBG* fasting blood glucose; *HbA1c* glycosylated hemoglobin A1c; *TC* total cholesterol; *TG* triglycerides; *LDL-C* low density lipoprotein cholesterol; *HDL-C* high density lipoprotein cholesterol; *BNP* B-type brain natriuretic peptide; *TnI* troponin I; *AST* aspartate aminotransferase; *ALT* alanine aminotransferase; *LA* left atrial diameter; *LVED* left ventricular diastolic diameter; *LVEF* left ventricular ejection fraction; *E/E’* Ratio of early diastolic mitral flow velocity to early diastolic mitral annular motion velocity

Hypolipidemic drugs: including statins, Bates, and niacin, mainly statins

### Association between coronary calcification and clinical risk factors

**Fig. 3:**
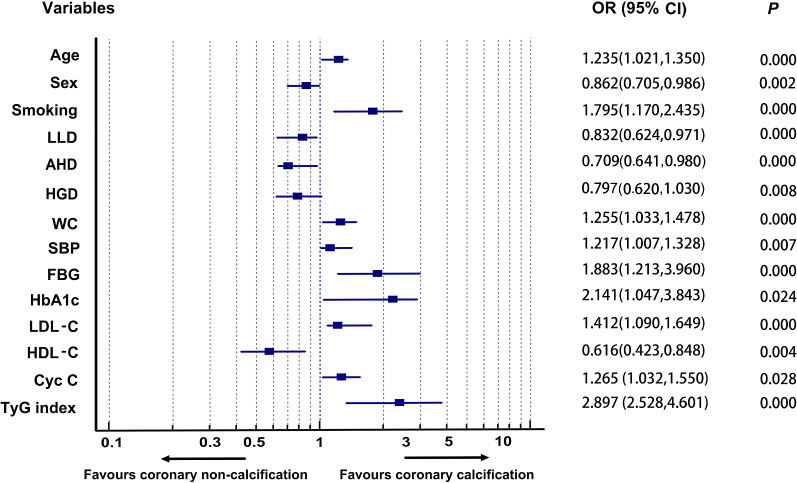
Forest plot demonstrated clinical risk factors significantly affecting coronary calcification. The association between clinical risk factors and coronary calcification degree was displayed in the forest plot. OR, Odd Ratio; CI: confidence interval, all *P* < 0.05%. Adjusted for age, gender, AHD (antihypertensive drugs), HGD (Hypoglycemic drugs), LLD (lipid-lowering drugs), body weight, BMI(body mass index), WC (waist circumference), SBP (systolic blood pressure), DBP (diastolic blood pressure), PPD(pulse pressure difference), HR (heart rate), FBG (fasting blood glucose), HbA1c (glycosylated hemoglobin A1c), LVEF, diabetes, hypertension, hyperlipidemia, smoking, drinking, white blood cell, TC(total cholesterol), LDL-C (low-density lipoprotein), HDL-C (high-density lipoprotein), Cr (creatinine), UA (uric acid), Cys C (Cystatin C), homocysteine, D-dimer, TnI (troponin I), and BNP (B-type natriuretic peptide) except for the stratified variables

Univariable logistic regression analysis indicated that age, smoking, hypertensive and diabetic history, antihypertensive drugs (AHD), hypoglycemic drugs (HGD), lipid-lowering drugs (LLD), weight, BMI, WC, SBP, DBP, pulse pressure difference (PPD), WBC, FBG, HbA1c, UA, TC, LDL-C, TG, HDL-C, cystatin C, homocysteine, TNI, BNP, HDL-C, and CACS, apart from sex, HR, Cr, ALT, AST, D-dimmer and drinking, were statistically associated with coronary calcification degree (Table 2). After adjusting for confounding factors, the correlation between coronary calcification degree and age, sex, smoking, LLD, AHD, HGD, WC, SBP, FBG, HbA1c, LDL-C, HDL-C, Cys C, and TyG index, is still consistent with the above results. Among them, the severity of coronary artery disease increased by 1.897 times for each unit of TyG index (Table 2, Fig. 3).

**Table 2:** Association between coronary calcification and clinical risk factors

Variables	Univariate analysis OR (95% CI)	*P*	Multivariate analysis for adjusted OR (95% CI)	*P*
Age	1.052 (1.040, 1.064)	0.000	1.235 (1.021,1.350)	0.000
Sex	0.941 (0.730, 1.213)	0.639	0.682 (0.705,0.986)	0.000
Weight	1.029 (1.014, 1.045)	0.000		
BMI	1.124 (1.096, 1.151)	0.000		
WC	1.064 (1.048, 1.080)	0.000	1.255 (1.033,1.478)	0.004
SBP	1.024 (1.017, 1.031)	0.000	1.217 (1.007,1.328)	0.007
DBP	1.008 (1.000, 1.016)	0.039		
Pulse pressure difference(PPD)	1.031 (1.023, 1.039)	0.000		
HR	1.008 (0.998,1.017)	0.102		
FBG	1.523 (1.412, 1.642)	0.000	1.883 (1.213,2.960)	0.000
HbA1c	1.336 (1.252, 1.424)	0.000	2.141 (1.047,3.843)	0.024
WBC	1.055 (1.008, 1.055)	0.022		
TC	0.834 (0.737, 0.943)	0.004		
Cr	1.003 (0.999, 1.008)	0.275		
TG	1.757 (1.498, 2.061)	0.000		0.000
LDL-C	1.184 (1.028, 1.363)	0.019	1.412 (1.090, 1.649)	0.000
HDL-C	0.480 (0.375, 0.614)	0.000	0.616 (0.423, 0.848)	0.004
BNP	1.000 (1.000, 1.002)	0. 001		
Cystatin C	1.899 (1.535, 2.350)	0.000	1.265 (1.032,1.550)	0.028
UA	1.003 (1.002, 1.004)	0.000		
Homocysteine	1.016 (1.003, 1.028)	0.013		
TyGindex	4.311 (3.413, 5.446)	0.000	2.897 (2.528, 4.601)	0.000
TnI	1.008 (1.002,1.015)	0.013		
D-dimmer	1.000 (1.000, 1.000)	0.552		
ALT	1.003 (0.998, 1.007)	0.235		
AST	1.001 (0.999, 1.002)	0.288		
Drinking	1.200 (0.919, 1.566)	0.180		
Smoking	1.811 (1.588, 2.066)	0.000	1.795 (1.17,2.435)	0.000
Diabetes history	1.855 (1.380, 2.494)	0.000		0.000
Antihypertensive drugs	0.647 (0.531, 0.747)	0.000	0.709 (0.641,0.980)	0.000
Lipid-lowering drugs	0.788 (0.656, 0.947)	0.011	0.832 (0.624,0.971)	0.000
Hypoglycemic drugs	0.904 (0.825, 0.991)	0.032	0.797 (0.62,1.03)	0.008
LA	1.003 (0.976, 1.031)	0.828		
LVED	1.006 (0.979, 1.033)	0.672		
EF	0.994 (0.980, 1.009)	0.458		
E/E`	1.014 (0.981, 1.048)	0.408		

Abbreviations as in Table 1

Lipid-lowering drugs: including statins, Bates and niacin, mainly statins

### Clinical features of coronary multivessel and non-multivessel diseases

**Fig. 4:**
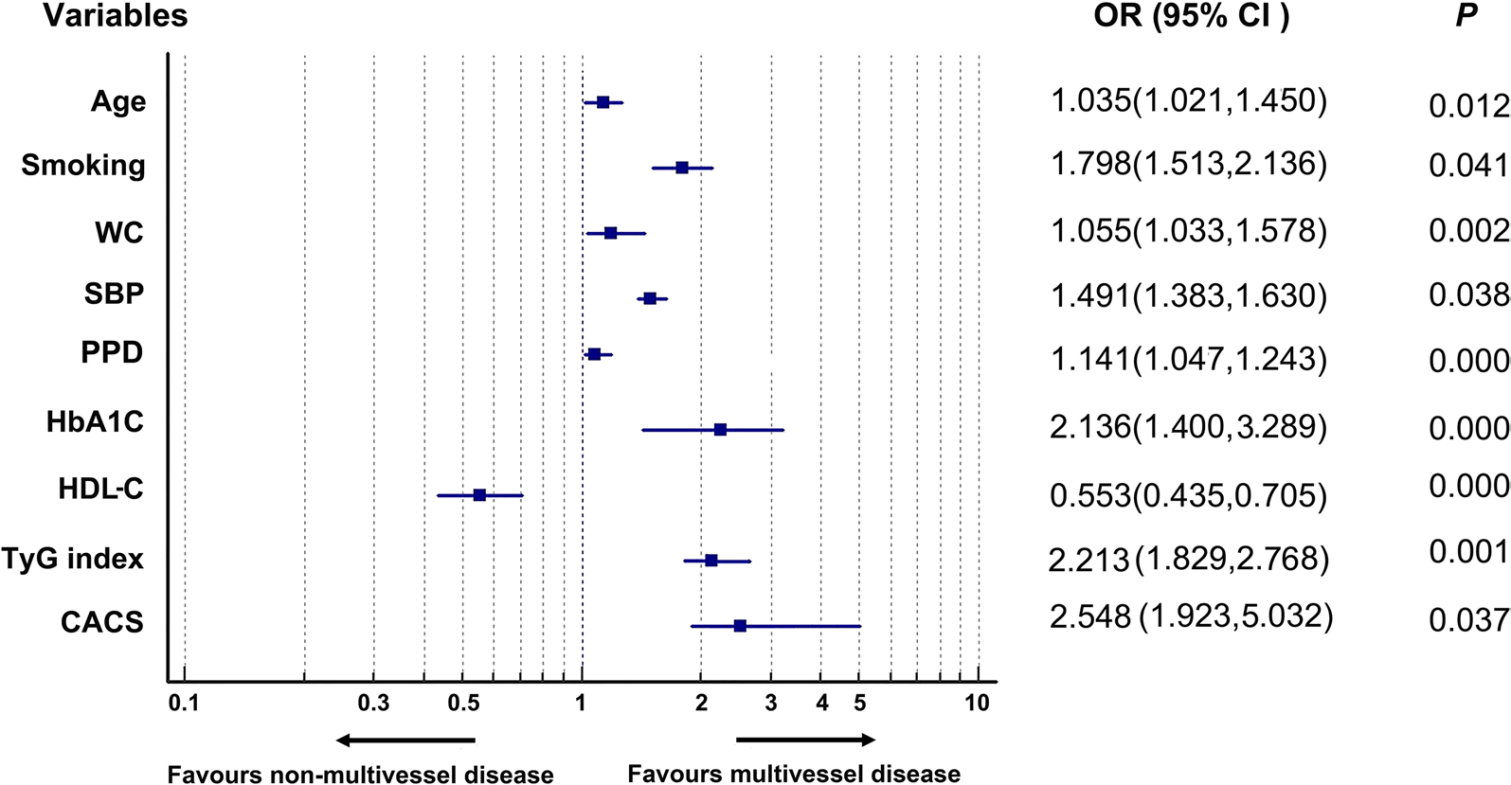
Forest plot displayed the clinical risk factors affecting the severity of coronary disease. Statistical methods and related abbreviations are the same as Fig. 3 (all *P* < 0.05%)

**Fig. 5:**
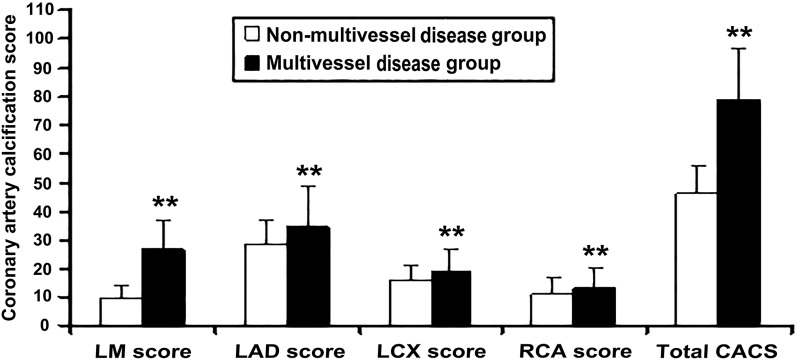
Comparison of CASC in each coronary branch between non-multivessel and multivessel disease groups. CASC**,** coronary calcification score; LM, left main artery; LAD, left anterior descending branch; LCX, left circumflex branch; RCA, right coronary artery. Coronary artery calcification score expressed as a categorical variable: CACS < 62.36 (median value); CACS ≥ 62.36. vs. non-multivessel group, *P < 0.05, **P < 0.01

According to the number and severity of coronary lesions, all subjects were divided into two groups: non-MVCD and MVCD groups. The comparison of baseline data between the MVCD and the non-MVCD groups is exposed in Table 3. As predicted, MVCD group had higher levels of age, SBP, DBP, PPD, BMI, WC, UA, cystatin C, FBG, HbA1c, TG, LDL-C, HDL-C, BNP, TnI, AST, and LA (*P* < 0.05) than those of the non-MVCD group (all *P* < 0.05) (Table 3). Nevertheless, no statistically significant differences were found in the other indicators: HR, WBC, Cr, TC, D-dimmer, homocysteine, ALT, LVED, LVEF, and E/E’ between the two groups (*P* > 0.05) (Table 3). After adjusting for age, gender, and other potential confounding factors, logistic regression analysis showed that age, smoking, waist circumference (WC), SBP, pulse pressure difference (PPD), HbA1c, HDL-C, TyG index, and CASC were the clinical risk factors affecting coronary artery disease (all *P* < 0.05) (Fig. 4). With regard to coronary calcification score, MVCD group had higher scores of LM, LAD, LCX, RCA, and total CACS than those of non-MVCD group (*P* < 0.001) (Fig. 5).

**Table 3:** Baseline characteristics of subjects stratified by coronary artery severity

Project	Non-MVCD group (n = 326)	MVCD group (n = 609)	*t or χ2*	*P*
General information				
Age (year)	56.70 ± 12.58	68.43 ± 12.57	0.014	0.000
Male sex [case (%)]	221 (67.79%)	432 (70.93%)	93.50	0.000
SBP (mmHg)	136.32 ± 18.50	153.76 ± 20.21	0.100	0.000
PPD (mmHg)	55.21 ± 15.41	71.44 ± 20.03	66.911	0.000
DBP (mmHg)	80.89 ± 11.24	82.01 ± 12.14	8.838	0.037
HR (bpm)	82.61 ± 50.64	80.83 ± 14.53	7.868	0.190
Past medical history				
Smoking [case (%)]	224 (68.71%)	468 (76.85%)	7.307	0.007
Drinking [case (%)]	174 (53.37%)	379 (62.23%)	6.535	0.011
Hypertension [case (%)]	180 (55.21%)	398 (65.35%)	9.25	0.024
Diabetes [case (%)]	127 (38.96%)	269 (44.14%)	21.44	0.000
Antihypertensive drugs [case (%)]	129 (39.26%)	181 (29.72%)	9.29	0.002
Hypoglycemic drugs [case (%)]	125 (38.34%)	171 (28.08%)	10.34	0.001
Hypolipidemic drugs [case (%)]	79 (24.23%)	103 (16.91%)	7.258	0.007
Anthropometric measurements				
Weight	70.04 ± 7.64	73.98 ± 8.59	19.74	0.000
Body mass index (BMI)	26.75 ± 3.51	29.22 ± 3.97	14.53	0.000
Waist circumstance (WC)	91.35 ± 8.30	98.97 ± 1.17	55.07	0.000
Laboratory indicators				
WBC (× 10^9^/L)	8.30 ± 2.77	8.63 ± 2.87	0.179	0.672
UA (umol/L)	349.98 ± 117.43	383.19 ± 131.29	18.38	0.000
Cr (umol/L)	80.99 ± 35.08	79.52 ± 28.00	1.195	0.274
Cystatin C (mg/L)	1.21 ± 0.66	1.63 ± 0.96	90.41	0.000
FBG (mmol/L)	6.79 ± 2.05	8.02 ± 2.93	67.22	0.000
HbAc1	7.29 ± 2.14	8.91 ± 2.81	89.391	0.000
TC (mmol/L)	4.33 ± 1.10	4.41 ± 1.12	0.612	0.104
TG (mmol/L)	1.86 ± 0.93	2.50 ± 1.34	69.72	0.000
LDL-c (mmol/L)	2.45 ± 0.97	2.70 ± 0.95	34.97	0.000
HDL-C (mmol/L)	1.35 ± 0.62	1.07 ± 0.52	82.41	0.000
BNP (pg/ml)	285.33 ± 469.47	343.87 ± 645.88	7.056	0.008
TnI (ng/ml)	13.74 ± 6.22	15.63 ± 7.93	6.516	0.013
D-dimer (ng/ml)	419.71 ± 544.93	434.32 ± 504.73	0.088	0.531
Homocysteine (umol/L)	16.70 ± 15.74	16.12 ± 9.86	6.915	0.285
AST(U/L)	48.19 ± 38.33	32.50 ± 26.20	24.040	0.000
ALT(U/L)	113.35 ± 75.00	94.24 ± 69.69	4.640	0.241
Echocardiography				
LA	37.41 ± 5.09	36.70 ± 4.92	0.239	0.002
LVED	48.64 ± 5.24	49.08 ± 4.91	0.743	0.052
LVEF	54.47 ± 9.47	54.25 ± 9.01	0.488	0.184
E/E’	9.50 ± 3.92	9.26 ± 4.14	0.301	0.591

Abbreviations as in Table 1

### Association between severity of coronary disease and TyG index

**Fig. 6:**
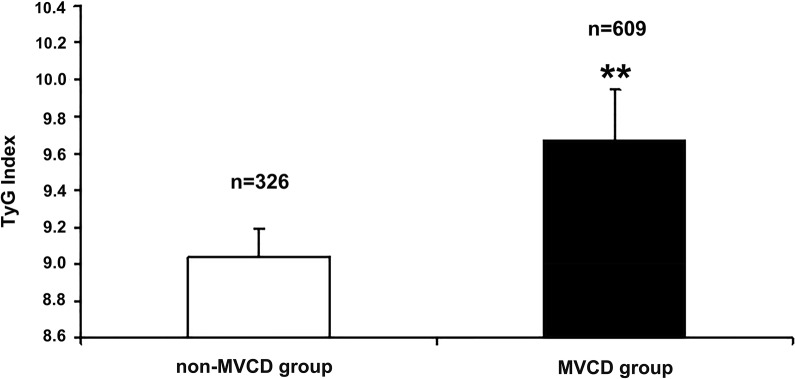
Comparison of TyG indexes between non-MVCD and MVCD groups. TyG index, triglyceride-glucose index; non-MVCD, Non-multivessel coronary disease; MVCD, multivessel coronary disease**.** Non-multivessel disease was defined as at least 50% stenosis in one major coronary artery. Multivessel disease was defined as at least two major vessels (≥ 2 mm diameter) from different territories with lesions deemed angiographically significant (≥ 50% stenosis). vs. non-MVCD, *P < 0.05, **P < 0.01

Compared with non-MVCD group, MVCD group had higher levels of TyG index (*P* > 0.001) (Fig. 6). As predicted, increasing TyG index quartiles was significantly correlated with the severity of coronary disease (Table 4). Taking quartile 1 (Q1) as the reference, multivariable logistic regression analysis demonstrated that the TyG index levels of Q2-Q4 increased the odds ratios for the severity of coronary disease in all groups including non-MVCD and MVCD groups (Table 4). Furthermore, after adjusting for confounding variables, including age, sex, blood pressure, serum lipid, and other risk factors, and taking drugs for diabetes, hypertension, and dislipidemia, these associations between TyG index quartiles and coronary disease severity also remained statistically significant (all *p* < 0.01) (Table 4). Compared with the hazard ratio (HR) in Q1 group, multivariate-adjusted hazard ratio of coronary disease also significantly increased with rising TyG index levels in Q2-Q4 groups (all *p* < 0.05): (1) In the group with ≤ 1 of coronary lesion, Q2: HR 2.91(95% CI 1.01–8.38); Q3: HR 3.47(95% CI 1.13–10.69); Q4: HR 4.88 (95% CI 1.64–14.50). (2) In the group with two coronary lesion, Q2: HR 2.29 (95% CI 1.21–4.35); Q3: 3.05 (95% CI 1.45–6.42); Q4: 4.11 (95% CI 1.99–8.46). (3) In the group with more than or equal to three coronary lesion, Q2: 1.61 (95% CI 1.02–3.19); Q3: 2.11 (95% CI 1.21–3.67); Q4: 2.66 (95% CI 1.38–5.13). Whether in mild coronary disease group or severe coronary disease group, especially in the mild coronary disease group, the TyG index contributed more to coronary artery disease (Table 4). Overall, as presented in Table 5, compared with non-MVCD, for each unit of TyG increase, the risk of multiple vascular disease increased by 1.213 times.

**Table 4:** Severity of coronary disease based on TyG index quartiles

Number of diseased coronary arteries	TyG index Quartiles	*β*	SE	Wald	Unadjusted OR (95% CI)	*P*	Adjusted OR (95% CI)	*P*
≤ 1	Q 1	Ref.						
	Q 2	1.74	0.50	12.07	5.67 (2.13, 15.08)	0.001	2.91 (1.01, 8.38)	0.047
	Q 3	2.18	0.53	16.78	8.824 (3.11, 25.01)	0.000	3.47 (1.13, 10.69)	0.030
	Q 4	2.97	0.49	37.23	19.47 (7.50, 50.54)	0.000	4.88 (1.64, 14.50)	0.004
2	Q 1	Ref.						
	Q 4	1.25	0.29	17.98	3.50 (1.96, 6.25)	0.000	2.29 (1.21, 4.35)	0.011
	Q 2	1.92	0.32	35.72	6.84 (3.64, 12.86)	0.000	3.05 (1.45, 6.42)	0.003
	Q 3	1.98	0.33	35.56	7.26 (3.78, 13.92)	0.000	4.11 (1.99, 8.46)	0.000
≥ 3	Q 1	Ref.						
	Q 2	1.08	0.29	13.43	2.94 (1.65, 5.24)	0.000	1.61 (1.02, 3.19)	0.017
	Q 4	1.10	0.25	19.20	3.01 (1.84, 4.93)	0.000	2.11 (1.21, 3.67)	0.008
	Q 3	1.42	0.30	22.09	4.12 (2.28, .43)	0.000	2.66 (1.38, 5.13)	0.023

Q1, 7.37–8.51; Q2, 8.82–8.99; Q3, 9.00–9.53; Q4, 9.54–11.504

*OR* Odd Ratio; *CI* confidence interval

**Table 5:** Evaluate the capacity of TyG index to predict coronary disease severity

Coronary disease severity	*β*	SE	Wald	Unadjusted HR (95% CI)	*P*	Adjusted HR (95% CI)	*P*
non-MVCD	− 6.546	0.887	54.352				
MVCD	0.794	0.097	66.717	3.251 (2.922, 3.617)	0.000	2.213 (1.829, 2.679)	0.001

*non-MVCD* non-multivessel coronary disease; *MVCD* multivessel coronary disease

### ROC curves and AUC estimated the severity of coronary disease

**Fig. 7:**
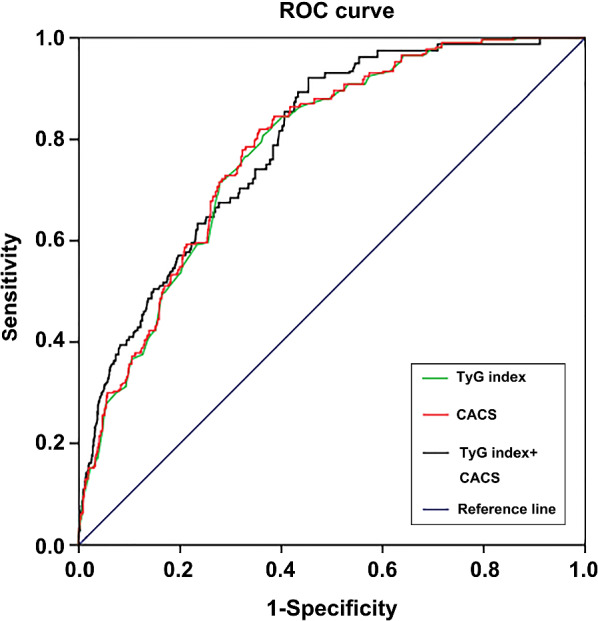
ROC curve analysis of TyG index or/and CASC to predict coronary disease severity. TyG index, triglyceride-glucose index; CASC, coronary artery calcium score

ROC curve was used to estimate the diagnostic value of CACS, TyG index and their combination for MVCD. As shown in Fig. 7, TyG index and CACS had approximate diagnostic ability for the severity of coronary disease in all subjects. The area under the ROC curve (AUC) of MVCD evaluated by TyG index was 0.780 (95% CI 0.756–0.804), with 60.7% sensitivity, 61.5% specificity, respectively; by CACS was 0.792 (95% CI 0.759–0.814), with 62.1% sensitivity and 64.7% specificity, respectively; by the combination of TyG index and CACS was 0.798 (95% CI 0.760–0.817), with 62.9% sensitivity and 67.3% specificity, respectively. The optimal cut-off value of TyG index for predicting MVCD was 9.0721 (sensitivity 60.4%, specificity 61.2%) (Fig. 7). According to the above results, TyG index had a very similar predictive strength and diagnostic capability for the severity of coronary disease to CASC. Surprisingly, the combination of the two methods did not increase the potential to predict coronary artery disease, only the sensitivity and specificity of prediction increased slightly.

### Relationship between TyG index quartiles and MACEs

**Fig. 8:**
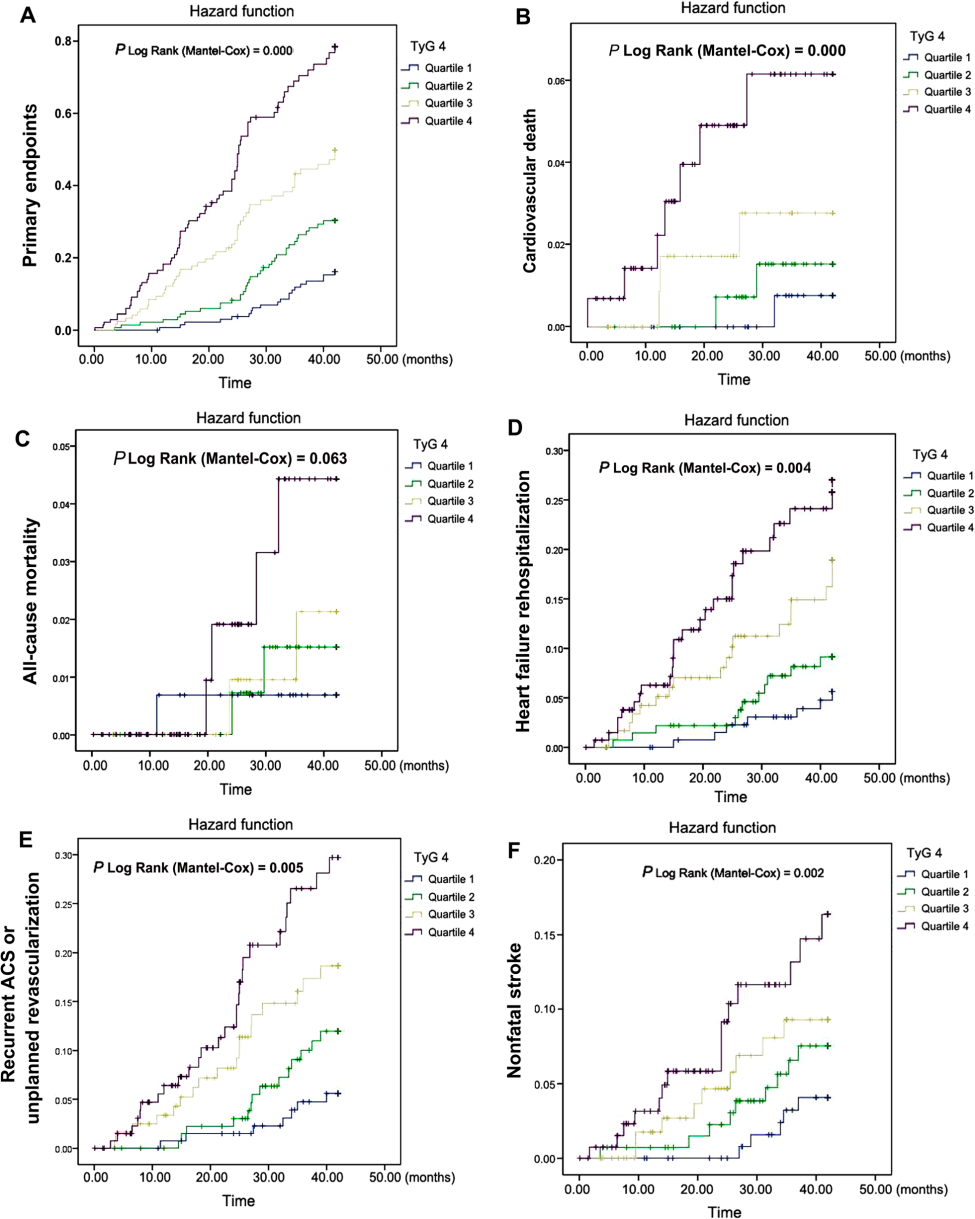
Cumulative hazard function of different clinical endpoints according to TyG indexes. Kaplan–Meier curves demonstrated the cumulative hazard function of the primary endpoint (**A**), cardiovascular death (**B**), all-cause death (**C**), heart failure rehospitalization (**D**), Hospitalization for non-fatal myocardial infarction, angina and unplanned revascularization (**E**), and non-fatal stroke (**F**) among the 4 groups based on the TyG index quartiles. TyG index quartile 1 (Q1), 7.37–8.51; quartile 2 (Q2), 8.82–8.99; quartile 3 (Q3), 9.00–9.53; quartile 4 (Q4), 9.54–11.504. Except for the primary endpoint, the comparison of other clinical endpoints between Q1-Q4 groups reached significant differences (*P* < 0.05)

Overall, the median follow-up time was 34.52 (interquartile range: from 26.50 to 42.00) months. Kaplan–Meier curves estimated cumulative incidences of each clinical event in major adverse cardiovascular events (MACEs) were shown in Fig. 8. The primary endpoints were the composite of all-cause mortality, cardiovascular death, unexpected rehospitalization of heart failure, recurrent ACS or unplanned revascularization, and non-fatal stroke. Kaplan Meier curve for the incidence of the primary endpoint and each component event stratified by the TyG index quartiles in all patients with ACS are presented in Fig. 8. Apart from all-cause death, the incidences of the primary endpoints, cardiac death, unexpected re-hospitalization of heart failure, recurrent ACS or unplanned revascularization, and non-fatal stroke in the Q4 group were significantly higher than those of the Q1- Q3 groups (*P* log-rank < 0.001). Obviously, with the increase of the quartiles of TyG index, the incidence of these events mentioned above increased gradually (all *P* < 0.01) (Fig. 8). On the contrary, although the incidence curve of all-cause death in each TyG quartile had a significant separation trend, the overall difference did not reach a statistically significant difference among all TyG index quartiles (log-rank test, *P* = 0.063).

## Discussion

In this study, we noticed a significant association between the TyG index, CACS, coronary lesion severity, and ASCVD outcomes in patients with ACS. Even after adjusting for as many potential confounding risk factors as possible, an independent association between the TyG index, MVCD, and adverse CV outcomes was retained. Moreover, the predictive intensity of TyG index for MVCD and coronary MACEs is similar to that of CACS. To the best of our knowledge, this is the first study to investigate the prognostic value of the TyG index in ACS patients with MVCD.

It was found that almost all components of metabolic syndrome are involved in coronary calcification and lesions in this study. Multivariate analysis demonstrated that the anthropometric indexes waist circumference, blood pressure (SBP), blood glucose (FBG, HbA1c), and blood lipid (LDL-C, HDL-C) levels of patients were closely related to TyG indexes (Table 1), coronary artery calcification (Table 2, Fig. 3), and the severity of coronary artery disease (Table 3, Fig. 4). As can be seen from the above, multivariate analysis displayed that BMI could not significantly impact the calcification and severity of coronary artery with ACS in this study. Although it is controversy that the impact intensity of BMI and waist circumstance on ASCVD [15, 20, 25], the results of this study still support that waist circumstance is a risk factor stronger than BMI for ASCVD. Our results are consistent with previous studies [25], Luan, et al. deem that BMI can only be used to assess general obesity, while WC can be used to assess central obesity (or abdominal obesity), more accurately epitomize the distribution of body fat, and better forecast the risk of obesity related ASCVD [25]. Mori H, et al. held the idea that coronary calcification tends to be higher in diabetic patients, which is associated with total plaque burden and is an independent risk factor for adverse outcomes [6]. Furthermore, calcification of coronary artery and other artery beds is more extensive in diabetic patients [26]. Multiple lines of evidence have supported the concept that vascular calcification is closely associated with HbA1c [26]. Consistent with this view, in this study, both fasting glucose and HbA1c were proportional to the degree of coronary artery calcification (Table 2, Fig. 3F). Further analysis showed that blood glucose had less effect on vascular disease than HbA1c (Figs. 3, 4). This reason may be explained by the use of hypoglycemic drugs, and the blood glucose value fluctuates greatly. FBG at a time point is less stable than HbA1c, which more stably reflects the average blood sugar level of the recent three months in these patients. The results observed in the above statistics in this study support the view that patients with a higher TyG indexes were more likely to develop obesity, hypertension, and diabetes than those with a lower TyG index [21].

As a traditional and unalterable risk factor for cardiovascular disease, age was closely related to TyG index and coronary artery calcification in this study, whether univariate analysis or multivariate analysis (Table 1–3, Figs. 3, 4). Even if the male sex exacerbated the coronary calcification (Table 2, Fig. 3), it ultimately did not affect the severity of coronary disease (Fig. 4). This is different from the view of Razavi AC, et al. that women are more likely to have lower CAC scores compared to men of the same age [27]. Meanwhile atherogenic lipoproteins are a modifiable risk factor for ASCVD. Traditionally, low density lipoprotein cholesterol (LDL-C) has been regarded a risk factor for ASCVD and a therapeutic target for atherosclerosis. However, there is increasing evidence that LDL-C may not be the best marker of cardiovascular disease and the risk of atherosclerotic lipoprotein [28]. In this study, HDL-C, LDL-C, antihypertensive drugs, hypoglycemic drugs, and lipid-lowering drugs (LLD) all had significant effects on CAC, whether univariate analysis or multivariate analysis (Table 2, Fig. 3). In contrast, univariate analysis showed that although HDL-C, LDL-C and Lipid-lowering drugs had significant effects on multivessel coronary artery disease. However, multivariate analysis confirmed that only LDL-C and HDL-C had a significant effect on coronary multivessel disease, and LDL-C has little impact on MVCD (*OR* = 1.141%, 95 CI 1.047, 1.443) (Table 3, Fig. 4). The reason may be related to the following factors: the use of hypolipidemic drugs (mainly refers to statins) interfered with the true effect of LDL-C on CAC; the increase of LDL-C in Chinese, especially in patients with metabolic syndrome, was not obvious, but mainly TG increased and HDL-C decreased. Some studies have found that non HDL-C may better reflect the overall burden of atherosclerotic lipoprotein than LDL-C [28]. High-density lipoprotein cholesterol (HDL-C) is recognized as a protective factor for ASCVD because previous studies have shown the inverse relationship between HDL-C and ASCVD events, particularly coronary heart disease [29]. Accordingly, HDL-C dysfunction might play a key role in the relationship between HDL-C and atherosclerosis [29]. Although some clinical trials claim that HDL-C alone plays a limited role in the protection of ASCVD [29], this study confirms that HDL-C is negatively correlated with both coronary calcification and MVCD (Table 3, Fig. 4F). In this study, we found that with the decrease of the use of lipid-lowering, antihypertensive and hypoglycemic drugs, the quartile of TyG index increased (Table 1). Furthermore, both univariate analysis and multivariate analysis suggested that the use of lipid-lowering, antihypertensive and hypoglycemic agents were strongly negatively correlated with CACS (Table 2, Fig. 3). Nevertheless, multivariate analysis showed that they had no significant effect on coronary disease severity (Fig. 4). From the above data, there are significant statistical differences in multiple traditional ASCVD risk factors among different TyG index, non-MCVD, and MCVD groups (Tables 1, 3, Figs. 3, 4). The association of the TyG index with coronary lesion might be partially mediated by these traditional cardiovascular risk factors and be interfered interfered by drugs for routine treatment of metabolic syndrome.

It is widely known that smoking is associated with a high risk of vascular calcification and cardiovascular disease [30]. In some prospective studies, longer duration to quit smoking is consistently associated with lower cardiovascular risk [30]. Studies have shown that current smokers are more likely to suffer from CAC than never smokers [30, 31]. As an independent risk factor for the development of coronary heart disease, smoking leads to inflammation, impaired endothelial function, platelet dysfunction, increased oxidative stress and atherosclerosis, all of which are related to the development and adverse events of ASCVD [31]. Although our study demonstrates that smoking is not the most important risk factor for the progression of CAC and MVCD in patients with ACS, it is a risk factor for coronary calcification and multivessel lesion second only to blood glucose and TyG index (Figs. 3, 4).

It is worth noting that in this study, we found that cystatin C increased with the increasing of TyG indexes (Table 1). Cystatin C is an independent risk factor for coronary calcification (Table 2, Fig. 3) and the risk of CAC increased 26.5% for each unit of cystatin C increased (Fig. 3). Although cystatin C values of MVCD group were significantly higher than those of non-MVCD group (Table 3), the multivariate analysis showed that cystatin C had no significant difference in the severity of coronary artery disease (Fig. 4). Researches in recent years have proved that cystatin C is an endogenous filtration marker, elevated concentration of cystatin C is associated with not only glomerular damage but also ASCVD [32]. ASCVD is an inflammatory disease characterized by extensive degradation of arterial wall matrix proteins. Cathepsin, a cysteine protease, plays a key role in extracellular matrix (ECM) remodeling and participates in cardiovascular diseases based on atherosclerosis [32]. As an endogenous inhibitor cystatin C, promising biomarkers in the diagnosis of coronary artery disease, it may favor proteolysis of extracellular matrix (ECM) in the pathogenesis of ASCVD by participating in inflammation and extracellular matrix remodeling [32]. The decline of renal function in CKD patients, independent of the identified risk factors for CVD, leads to calcium and phosphorus metabolism disorders and inflammation, which may be the cause of CAC progression [33].

TyG index can be calculated by measuring only two laboratory indicators of triglyceride and fasting blood glucose. So it is convenient and less expensive to detect, but it is of great significance for guiding clinical work that TyG index has also been proved to be a better predictor of cardiovascular disease than FBG and HbA1c [21]. Consistent with the previous research, it is found that TyG index has stronger predictive ability for coronary calcification than FBG and HbA1c in this study (Table 2, Fig. 3). With the increase of TyG index, the total CACS and the CACS of each coronary artery branch (LM, LAD, LCX, and RCA) in each patient with ACS also gradually increased (all *P* < 0.05) (Fig. 2). Consistent with previous studies [13, 15, 17, 20], we also found that even after adjusting for as many potential confounding factors as possible, the TyG index as an independent risk factor was still associated with coronary severity(Tables 4, 5, Figs. 4, 7). Compared with non-MVCD, MVCD had higher TyG index and CASC in each coronary branch (LM, LAD, LCX, and RCA) (*P* < 0.01) (Figs. 5, 6), which was more in line with previous studies [34] that the LAD score is relatively higher than the scores of other parts of the blood vessels (Fig. 5), reflecting from the side that LAD may be the more common part of coronary calcification**. **Sugiyama insisted that a high proportion of spot-like calcification, often located in left anterior descending coronary arteries (LAD), are the most common plaque type in ACS [11]. Consistent with the previous findings, our study confirmed that LAD had the highest coronary artery calcification score in all major branches of the coronary artery, regardless of whether it was stratified by TyG index or grouped by severity of coronary artery disease (Figs. 1, 2, 5). Recently, Lee EY and Thai PV have found that the TyG index is associated to coronary artery stenosis through clinical studies [35, 36]. Consistent with the previous research results, this study displayed that the higher the TyG index, the greater the number of coronary artery stenosis, the more serious the disease (Table 4, Fig. 4). Moreover, TyG index is independent risk factors for MVCD (*OR* = 2.213, 95%CI 1.829, 2.768, *P* = 0.001), that is, for each unit of TyG increase, the risk of multiple vascular disease increased by 1.213 times (Table 5, Fig. 4). Especially, in the mild coronary disease group, the TyG index contributed more to coronary artery disease (Table 4). Not coincidentally, Park et al. suggested that TyG index was an effective marker for early detecting subclinical coronary atherosclerosis even in the absence of traditional CV risk factors [37]. Compared with TyG index, CACS had slightly stronger correlation and prediction ability for MVCD (*OR* = 2.548, 95% CI 1.923, 5.032, *P* = 0.037) (Fig. 4).

Although the area under the curve of ROC curve (AUC) of TyG index for the diagnosis of MVCD is only 0.792, which is extraordinarily close to the AUC (0.780) of CACS in the same drawing (Fig. 7). Jiao Y and Wang L, et al. declared the view that TyG index is independent of known cardiovascular risk factors to predict future MACEs in patients with diabetes and ACS [17, 18]. In this study, with the increase of the quartiles of TyG index, the incidence of the MACEs, including cardiac death, unexpected re-hospitalization of heart failure, recurrent ACS or unplanned revascularization, and non-fatal stroke, except for all-cause death, gradually increased in the Q1-Q4 groups (all *P* log-rank < 0.01) (Fig. 8). Our study provides additional information to support previous studies, suggesting the clinical significance of TyG index in predicting cardiovascular risk and MACE in patients with ACS (Fig. 8).

To sum up, this study confirmed the role of TyG index in Chinese patients with coronary calcification and MVCD. As mentioned above, TyG index could not only predict the degree of coronary calcification and disease, but also effectively forecast the MACE of ASCVD in patients with ACS. This study suggested that TyG index could be used as an independent risk factor to substitute CACS for predicting the progression and MACEs of ASCVD. During the clinical practice, combining TyG index with routine risk factors of ASCVD may significantly promote the accuracy of clinical risk prediction of cardiovascular disease prognosis. Therefore, screening high-risk cardiovascular population with TyG index is helpful to timely prevent and control the occurrence and development of ASCVD, reduce coronary multivessel disease and MACEs, and improve the survival and the long-term prognosis of patients with ACS patients.

## Limitations

There are several limitations worth considering in this study. Firstly, limited to the resolution and technical bottleneck of CCTA, the early micro-calcification of coronary atherosclerotic lesions is difficult to identify. Secondly, the formation of early atherosclerotic plaque may not involve calcification, or some patients do not have obvious atherosclerotic plaque, such as coronary spasm or microcirculation disorders, with 0 or low CACS may also suffer from ACS. Thirdly, there are always residual confounding factors that influence the final results of observational studies. Fourthly, we could not perform time-dependent analysis because some parameters such as FBG and blood lipid change with time. We only collected the observed values at one time point. Fifthly, the statistical results may be biased because we have not fully considered the intensity, time and outcome of anti-diabetic therapy, lipid-lowering, antihypertensive, and anti heart failure treatment, et al. Sixthly, some traditional risk factors for coronary heart disease, such as gender and abnormal LDL, were not associated with MVCD, which may be related to small sample size, the presence of multiple risk factors, and the use of different types of drugs. In the future, multi-center, large-sample, prospective studies are still expected to explore further the relationship between the TyG index, CACS, and MVCD. Finally, it is concluded that there may be racial differences. The current results may not be extended to other nationalities because the participants in our study are Chinese.

## Conclusion

In brief, almost all components of metabolic syndrome are involved in coronary calcification and lesions in this study, and triglyceride glucose product index could completely substitute for CACS as a reliable, practical, and independent indicator for predicting the severity and prognosis of MVCD in patients with ACS.

## Data Availability

The datasets used and analysed during the current study are available from the corresponding author on reasonable request.

## Abbreviations

ACS: Acute coronary syndrome
AHD: Antihypertensive drugs
ALT: Alanine aminotransferase
ASCVD: Arteriosclerotic cardiovascular disease
AST: Aspartate aminotransferase
BNP: Brain natriuretic peptide
CAC: Coronary artery calcification
CACS: Coronary artery calcification score
CCTA: Coronary computed tomography angiography
CPR: Surface reconstruction
Cr: Creatinine
Cys C: Cystatin C
DBP: Diastolic blood pressure
FBG: Fasting blood-glucose
HbA1c: Glycosylated Hemoglobin A1c
Hcy: Homocysteine
HDL-C: High-density lipoprotein cholesterol
HGD: Hypoglycemic drugs
HIEC: Hyperinsulinemic-euglycemic clamp
HOMA: Homeostasis model assessment
HR: Heart rate
IR: Insulin resistance
IVUS: Intra-vascular ultra sound
LAD: Left anterior descending coronary artery
LA: Left atrial diameter
LCX: Left circumflex artery
LDL-C: Low-density lipoprotein cholesterol
LLD: Lipid-lowering drugs
LM: Left main artery
LVED: Left ventricular end-diastolic volume
LVEF: Left ventricular ejection fraction
MACEs: Major adverse cardiovascular events
MIP: Maximum intensity projection
MPR: Multi-planar reconstruction
MVCD: Multivessel coronary disease
non-MVCD: Non-multivessel coronary disease
NSTEMI: Non-ST-elevation myocardial infarction
OCT: Optical coherence tomography
PPD: Pulse pressure difference
RCA: Right coronary artery
SBP: Systolic blood pressure
SSD: Surface shaded display
STEMI: STEMIST-elevation myocardial infarction
T2DM: Type 2 diabetes
TC: Total cholesterol
TG: Triglyceride
TnI: Troponin I
TyG: Triglyceride glucose
UA: Uric acid
UAP: Unstable angina pectoris
VR: Volume reproduction
WBC: White blood cell

## References

[1] Zhao X, Wang Y, Chen R, Li J, Zhou J, Liu C, Zhou P, Sheng Z, Chen Y, Song L, Zhao H, Yan H (2021). Triglyceride glucose index combined with plaque characteristics as a novel biomarker for cardiovascular outcomes after percutaneous coronary intervention in ST-elevated myocardial infarction patients: an intravascular optical coherence tomography study. Cardiovasc Diabetol.

[2] Luo E, Wang D, Yan G, Qiao Y, Liu B, Hou J, Tang C (2019). High triglyceride-glucose index is associated with poor prognosis in patients with acute ST-elevation myocardial infarction after percutaneous coronary intervention. Cardiovasc Diabetol.

[3] Kurihara O, Takano M, Yamamoto E, Yonetsu T, Kakuta T, Soeda T, Yan BP, Crea F, Higuma T, Kimura S, Minami Y, Adriaenssens T, Boeder NF, Nef HM, Kim CJ, Thondapu V, Kim HO, Russo M, Sugiyama T, Fracassi F, Lee H, Mizuno K, Jang IK (2020). Seasonal variations in the pathogenesis of acute coronary syndromes. J Am Heart Assoc.

[4] Shiyovich A, Shlomo N, Cohen T, Iakobishvili Z, Kornowski R, Eisen A (2020). Temporal trends of patients with acute coronary syndrome and multivessel coronary artery disease - from the ACSIS registry. Int J Cardiol.

[5] Zaman MO, Mojadidi MK, Elgendy IY (2019). Revascularization strategies for patients with myocardial infarction and multi-vessel disease: a critical appraisal of the current evidence. J Geriatr Cardiol.

[6] Mori H, Torii S, Kutyna M, Sakamoto A, Finn AV, Virmani R (2018). Coronary artery calcification and its progression: what does it really mean?. JACC Cardiovasc Imaging.

[7] Nakahara T, Dweck MR, Narula N, Pisapia D, Narula J, Strauss HW (2017). Coronary artery calcification: from mechanism to molecular imaging. JACC Cardiovasc Imaging.

[8] Koulaouzidis G, Charisopoulou D, Jenkins PJ, Koulaouzidis A, McArthur T (2013). Prevalence of non-calcified coronary plaque in patients with calcium score of 0: the silent enemy. Angiology.

[9] Koulaouzidis G, Charisopoulou D, Maffrett S, Tighe M, Jenkins PJ, McArthur T (2013). Differences in clinical profile of individuals with severe and markedly elevated coronary artery calcification detected by electron beam computed tomography. Angiology.

[10] Henein MY, Koulaouzidis G, Granasen G, Wiklund U, Guerci A, Schmermund A (2013). The natural history of coronary calcification: a meta-analysis from St Francis and EBEAT trials. Int J Cardiol.

[11] Sugiyama T, Yamamoto E, Fracassi F, Lee H, Yonetsu T, Kakuta T, Soeda T, Saito Y, Yan BP, Kurihara O, Takano M, Niccoli G, Crea F, Higuma T, Kimura S, Minami Y, Ako J, Adriaenssens T, Boeder NF, Nef HM, Fujimoto JG, Fuster V, Finn AV, Falk E, Jang IK (2019). Calcified plaques in patients with acute coronary syndromes. JACC Cardiovasc Interv.

[12] Patel J, Pallazola VA, Dudum R, Greenland P, McEvoy JW, Blumenthal RS, Virani SS, Miedema MD, Shea S, Yeboah J, Abbate A, Hundley WG, Karger AB, Tsai MY, Sathiyakumar V, Ogunmoroti O, Cushman M, Savji N, Liu K, Nasir K, Blaha MJ, Martin SS, Al RM (2021). Assessment of coronary artery calcium scoring to guide statin therapy allocation according to risk-enhancing factors: the multi-ethnic study of atherosclerosis. JAMA Cardiol.

[13] Won KB, Park EJ, Han D, Lee JH, Choi SY, Chun EJ, Park SH, Han HW, Sung J, Jung HO, Chang HJ (2020). Triglyceride glucose index is an independent predictor for the progression of coronary artery calcification in the absence of heavy coronary artery calcification at baseline. Cardiovasc Diabetol.

[14] Reith S, Milzi A, Lemma ED, Dettori R, Burgmaier K, Marx N, Burgmaier M (2019). Intrinsic calcification angle: a novel feature of the vulnerable coronary plaque in patients with type 2 diabetes: an optical coherence tomography study. Cardiovasc Diabetol.

[15] Park K, Ahn CW, Lee SB, Kang S, Nam JS, Lee BK, Kim JH, Park JS (2019). Elevated TyG Index predicts progression of coronary artery calcification. Diabetes Care.

[16] Williams MC, Moss AJ, Dweck M, Adamson PD, Alam S, Hunter A, Shah ASV, Pawade T, Weir-McCall JR, Roditi G, van Beek EJR, Newby DE, Nicol ED (2019). Coronary artery plaque characteristics associated with adverse outcomes in the SCOT-HEART study. J Am Coll Cardiol.

[17] Wang L, Cong HL, Zhang JX, Hu YC, Wei A, Zhang YY, Yang H, Ren LB, Qi W, Li WY, Zhang R, Xu JH (2020). Triglyceride-glucose index predicts adverse cardiovascular events in patients with diabetes and acute coronary syndrome. Cardiovasc Diabetol.

[18] Jiao Y, Su Y, Shen J, Hou X, Li Y, Wang J, Liu B, Qiu D, Sun Z, Chen Y, Xi Q, Shen M, Fu Z (2022). Evaluation of the long-term prognostic ability of triglyceride-glucose index for elderly acute coronary syndrome patients: a cohort study. Cardiovasc Diabetol.

[19] Simental-Mendía LE, Ortega-Pacheco CJ, García-Guerrero E, Sicsik-Aragón MA, Guerrero-Romero F, Martínez-Aguilar G (2021). The triglycerides and glucose index is strongly associated with hepatic steatosis in children with overweight or obesity. Eur J Pediatr.

[20] Ma X, Dong L, Shao Q, Cheng Y, Lv S, Sun Y, Shen H, Wang Z, Zhou Y, Liu X (2020). Triglyceride glucose index for predicting cardiovascular outcomes after percutaneous coronary intervention in patients with type 2 diabetes mellitus and acute coronary syndrome. Cardiovasc Diabetol.

[21] Hu C, Zhang J, Liu J, Liu Y, Gao A, Zhu Y, Zhao Y (2020). Discordance between the triglyceride glucose index and fasting plasma glucose or HbA1c in patients with acute coronary syndrome undergoing percutaneous coronary intervention predicts cardiovascular events: a cohort study from China. Cardiovasc Diabetol.

[22] Park B, Lee HS, Lee YJ (2021). Triglyceride glucose (TyG) index as a predictor of incident type 2 diabetes among nonobese adults: a 12-year longitudinal study of the Korean Genome and Epidemiology Study cohort. Transl Res.

[23] Zheng Q, Jiang J, Huo Y, Chen D (2019). Genetic predisposition to type 2 diabetes is associated with severity of coronary artery disease in patients with acute coronary syndromes. Cardiovasc Diabetol.

[24] Almendro-Delia M, Seoane García T, Villar Calle P, García González N, Lorenzo López B, Cortés FJ, García Del Río M, Ruiz García MDP, Hidalgo Urbano RJ, García-Rubira JC (2021). Prevalence and clinical significance of totally occluded infarct-related arteries in patients with non-ST-segment elevation acute coronary syndromes. Int J Cardiol.

[25] Luan H, Song Y, Cao L, Wang P, Zhu D, Tian G (2021). Gender differences in the relationship of waist circumference to coronary artery lesions and one-year re-admission among coronary artery disease patients with normal body mass index. Diabetes Metab Syndr Obes.

[26] Yahagi K, Kolodgie FD, Lutter C, Mori H, Romero ME, Finn AV, Virmani R (2017). Pathology of human coronary and carotid artery atherosclerosis and vascular calcification in diabetes mellitus. Arterioscler Thromb Vasc Biol.

[27] Razavi AC, Wong N, Budoff M, Bazzano LA, Kelly TN, He J, Fernandez C, Lima J, Polak JF, Mongraw-Chaffin M, deFilippi C, Szklo M, Bertoni AG, Blumenthal RS, Blaha MJ, Whelton SP (2021). Predicting long-term absence of coronary artery calcium in metabolic syndrome and diabetes: the MESA study. JACC Cardiovasc Imaging.

[28] Björnsson E, Thorleifsson G, Helgadóttir A, Guðnason T, Guðbjartsson T, Andersen K, Grétarsdóttir S, Ólafsson Í, Tragante V, Ólafsson ÓH, Jónsdóttir B, Eyjólfsson GI, Sigurðardóttir Ó, Thorgeirsson G, Guðbjartsson DF, Thorsteinsdóttir U, Hólm H, Stefánsson K (2020). Association of genetically predicted lipid levels with the extent of coronary atherosclerosis in icelandic adults. JAMA Cardiol.

[29] Hirata A, Kakino A, Okamura T, Usami Y, Fujita Y, Kadota A, Fujiyoshi A, Hisamatsu T, Kondo K, Segawa H, Sawamura T, Miura K, Ueshima H, SESSA Research Group (2020). The relationship between serum levels of LOX-1 ligand containing ApoAI as a novel marker of dysfunctional HDL and coronary artery calcification in middle-aged Japanese men. Atherosclerosis..

[30] Lee MJ, Park JT, Chang TI, Joo YS, Yoo TH, Park SK, Chung W, Kim YS, Kim SW, Oh KH, Kang SW, Choi KH, Ahn C, Han SH (2021). Smoking cessation and coronary artery calcification in CKD. Clin J Am Soc Nephrol.

[31] Oshunbade AA, Kassahun-Yimer W, Valle KA, Hamid A, Kipchumba RK, Kamimura D, Clark D, White WB, DeFilippis AP, Blaha MJ, Benjamin EJ, O'Brien EC, Mentz RJ, Rodriguez CJ, Fox ER, Butler J, Keith RJ, Bhatnagar A, Marie Robertson R, Correa A, Hall ME (2021). Cigarette smoking, incident coronary heart disease, and coronary artery calcification in black adults: the Jackson heart study. J Am Heart Assoc.

[32] Wu H, Du Q, Dai Q, Ge J, Cheng X (2018). Cysteine protease cathepsins in atherosclerotic cardiovascular diseases. J Atheroscler Thromb.

[33] Bundy JD, Chen J, Yang W, Budoff M, Go AS, Grunwald JE, Kallem RR, Post WS, Reilly MP, Ricardo AC, Rosas SE, Zhang X, He J, CRIC Study Investigators (2018). Risk factors for progression of coronary artery calcification in patients with chronic kidney disease: the CRIC study. Atherosclerosis..

[34] Bittner DO, Mayrhofer T, Bamberg F (2017). Impact of coronary calcification on clinical management in patients with acute chest pain. Circ Cardiovasc Imaging.

[35] Lee EY, Yang HK, Lee J (2016). Triglyceride glucose index, a marker of insulin resistance, is associated with coronary artery stenosis in asymptomatic subjects with type 2 diabetes. Lipids Health Dis.

[36] Thai PV, Tien HA, Van Minh H (2020). Triglyceride glucose index for the detection of asymptomatic coronary artery stenosis in patients with type 2 diabetes. Cardiovasc Diabetol.

[37] Park GM, Cho YR, Won KB, Yang YJ, Park S, Ann SH, Kim YG, Park EJ, Kim SJ, Lee SG, Yang DH, Kang JW, Lim TH, Kim HK, Choe J, Lee SW, Kim YH (2020). Triglyceride glucose index is a useful marker for predicting subclinical coronary artery disease in the absence of traditional risk factors. Lipids Health Dis.

